# Ancient duplication and functional differentiation of phytochelatin synthases is conserved in plant genomes

**DOI:** 10.1093/hr/uhae334

**Published:** 2024-11-26

**Authors:** Mingai Li, Jiamei Yu, Silvia Sartore, Erika Bellini, Daniela Bertoldi, Stefania Pilati, Alessandro Saba, Roberto Larcher, Luigi Sanità di Toppi, Claudio Varotto

**Affiliations:** Research and Innovation Centre, Fondazione Edmund Mach, via Mach 1, 38098 San Michele all’Adige, Trento, Italy; NBFC, National Biodiversity Future Center, Palermo 90133, Italy; Research and Innovation Centre, Fondazione Edmund Mach, via Mach 1, 38098 San Michele all’Adige, Trento, Italy; Research and Innovation Centre, Fondazione Edmund Mach, via Mach 1, 38098 San Michele all’Adige, Trento, Italy; Dipartimento di Biologia, Università di Pisa, via Luca Ghini 13, 56126 Pisa, Italy; Food Characterization and Processing Department, Technology Transfer Centre, Fondazione Edmund Mach, via Mach 1, 38098 San Michele all’Adige, Trento, Italy; Research and Innovation Centre, Fondazione Edmund Mach, via Mach 1, 38098 San Michele all’Adige, Trento, Italy; Dipartimento di Patologia Chirurgica, Medica, Molecolare e dell’Area Critica, Università di Pisa, via Roma 67, 56126 Pisa, Italy; Center for Instrument Sharing of the University of Pisa (CISUP), Lungarno Pacinotti, 43/44, 56126 Pisa, Italy; Food Characterization and Processing Department, Technology Transfer Centre, Fondazione Edmund Mach, via Mach 1, 38098 San Michele all’Adige, Trento, Italy; Dipartimento di Biologia, Università di Pisa, via Luca Ghini 13, 56126 Pisa, Italy; Research and Innovation Centre, Fondazione Edmund Mach, via Mach 1, 38098 San Michele all’Adige, Trento, Italy; NBFC, National Biodiversity Future Center, Palermo 90133, Italy

## Abstract

Despite the paramount importance in metal(loid) detoxification by *phytochelatin synthase* (*PCS*) genes, no comprehensive analysis of their evolutionary patterns has been carried out in land plants in general and in crops in particular. A phylogenetic large-scale analysis of gene duplication in angiosperms was carried out followed by *in vitro* recombinant protein assays as well as complementation analysis (growth, thiol-peptides, elements) of *Arabidopsis cad1–3* mutant with four representative *PCS* genes from two model crop species, *Malus domestica* and *Medicago truncatula*. We uncovered a so far undetected ancient tandem duplication (D duplication) spanning the whole core eudicotyledon radiation. Complementation with *PCS* genes from both D-subclades from *M. domestica* and *M. truncatula* displayed clear *in vivo* conservation of the differences between D1 and D2 paralogous proteins in plant growth, phytochelatin, and glutathione pools, as well as element contents under metal(loid) stress. *In vitro* recombinant PCS analysis identified analogous patterns of differentiation, showing a higher activity of D2 *PCS* genes, so far largely overlooked, compared to their paralogs from the D1 clade. This suggests that in many other crop species where the duplication is present, the D2 copy might play a significant role in metal(loid) detoxification. The retention of both *PCS* paralogs and of their functional features for such long divergence time suggests that *PCS* copy number could be constrained by functional specialization and/or gene dosage sensitivity. These results uncover the patterns of *PCS* evolution in plant genomes and of functional specialization of their paralogs in the genomes of two important model crops.

## Introduction

Phytochelatins (PCn) are cysteine-rich peptides, widely present not only in plants, but also in other organisms, with important functions in metal(loid) detoxification and homeostasis [1–3]. Their general structure (γ-Glu-Cys)n- X (where n = 2–5, X is generally glycine) [4] is the result of non-ribosomal synthesis catalyzed by the enzyme phytochelatin synthase (PCS), which extends PC polypeptide chains by adding glutathione units from C-terminus to N-terminus through a transpeptidation reaction. In the presence of heavy metals blocking the thiol groups of glutathione (GSH) or analogous thiol peptides, PCS synthesizes PCn containing up to 11 γ-Glu-Cys monomers [5]. PCn chelate the free metal ions (and the metalloid arsenic in the form of arsenite, AsIII) through the thiol group of cysteine, and the complexes of PCn::metal ions formed in the cytosol are transported by ATP-binding cassette (ABC) transporters to vacuoles, thus reducing the toxicity associated to accumulation of metal ions in the cytosol and regulating metal ion homeostasis [6, 7].

While most of the past studies were limited to the characterization of single *PCS* genes from each species, a handful of papers addressed the broader evolutionary question of the functional specialization of different *PCS* paralogs from the same species. Not surprisingly, the most studied *PCS* paralogs are *AtPCS1* and *AtPCS2* from *Arabidopsis thaliana* (L.) Heynh., which have been reported to have evolved marked functional differences. *AtPCS1*, the first gene for which a knockout mutant has been isolated and characterized [8], is nearly ubiquitously and constitutively expressed throughout *Arabidopsis* organs and developmental stages and plays the major role in the detoxification of heavy metals and arsenic as well as a likely role in plant innate immunity [9]. On the other hand, *AtPCS2* does not play a role as important as *AtPCS1* despite it is constitutively active and can partly rescue cadmium hypersensitivity of *cad1–3*, *AtPCS1* most commonly used loss-of-function mutant [10, 11].

Although *AtPCS1* and *AtPCS2* represent quite an extreme case of functional diversification among *PCS* paralogs, they are nonetheless emblematic of the tendency of the other handful of *PCS* paralogs characterized so far to assume different and sometimes complementary metal specificities in their host species, often through a combination of sub- and/or neofunctionalization. Given the high conservation of the N-terminal domain, it has been proposed that their fast-evolving C-terminus may mediate part of the functional variability among different PCS by defining the range of metals able to activate the enzyme, enhancing the core catalysis exerted by free heavy metals, increasing thermal stability, preventing overactivation and damaging of the catalytic N-terminal domain as well as having metallochaperone- and metallothionein-like functions [3, 12–14]. For instance, detailed work on AtPCS1 could pinpoint different regions of the C-terminal domain responsible to specific activation by different metals [10, 15]. As of today, however, it has not been investigated whether the homologous regions of AtPCS2 share the same functions. Another case of the functional differentiation between *PCS* paralogs resulting from C-terminal divergence comes from the family Fabacea. In *Lotus japonicus* (Regel) K.Larsen, the differences between the C-terminal domains of LjPCS1 and LjPCS3, two of the three *PCS* copies present in this species’ genome, were suggested to be responsible for the copy-specific activation by different sets of metals of these paralogs [16]. In another dicotyledon species, *Morus notabilis* C.K.Schneid., overexpression of *MnPCS1* and *MnPCS2* in wild-type (WT) *Arabidopsis* indicated a possible functional differentiation of the paralogs [17].

Also, the two *PCS* paralogs existing in the rice genome, *OsPCS1* and *OsPCS2* [18–22], present different specificities toward arsenic and cadmium, and provide distinct contributions to their detoxification [19, 22]. In another monocotyledon, the perennial rhizomatous grass *Arundo donax* L., three functional *PCS* paralogs displayed clear differentiation in their transcriptional responsiveness as well enzymatic activity in response to Cd exposure [23]. Along this sparse information on the functional specialization of different *PCS* paralogs from the same species, several partial PCS phylogenies focusing more or less closely on the clade of interest were carried out, revealing a patchy pattern of duplications in different plant families [10, 17, 23, 24]. Based on the limited number of fully sequenced genomes available at the time, Kühnlenz et al. [10] hypothesized that ‘the presence of at least two *PCS* genes appears to be a general feature of plant genomes’. Up to now, however, a unifying and broad phylogenetic overview of *PCS* duplication history in angiosperms to corroborate this hypothesis is missing. In the current work, we carried out a comprehensive phylogenetic reconstruction of PCS in angiosperms leveraging on the considerable number of high-quality genomes of crops and their relatives currently available in Phytozome 13 [25]. We uncovered the remnants of a deep duplication distributed across distantly related families, in addition to the two independent, family-specific duplications already reported for Brassicaceae, Poaceae, and Fabaceae [10, 23]. Given the long time for which several *PCS* paralogs resulting from the ancient duplication were maintained, we asked whether copies from the same clade share any functional specialization or if they evolved different functional specializations toward metals in every clade. For this reason, we isolated and functionally characterized both *in vitro* and *in vivo* paralogs from one species of Fabaceae (*Medicago truncatula* Gaertn.) and Rosaceae (*Malus domestica* (Suckow) Borkh.) providing the first overview of the patterns of evolution and functional specialization of PCS and, more in general, of metal detoxification in flowering plants.

## Results

### Identification of large-scale, clade-specific PCS duplications in land plants

**Figure 1:**
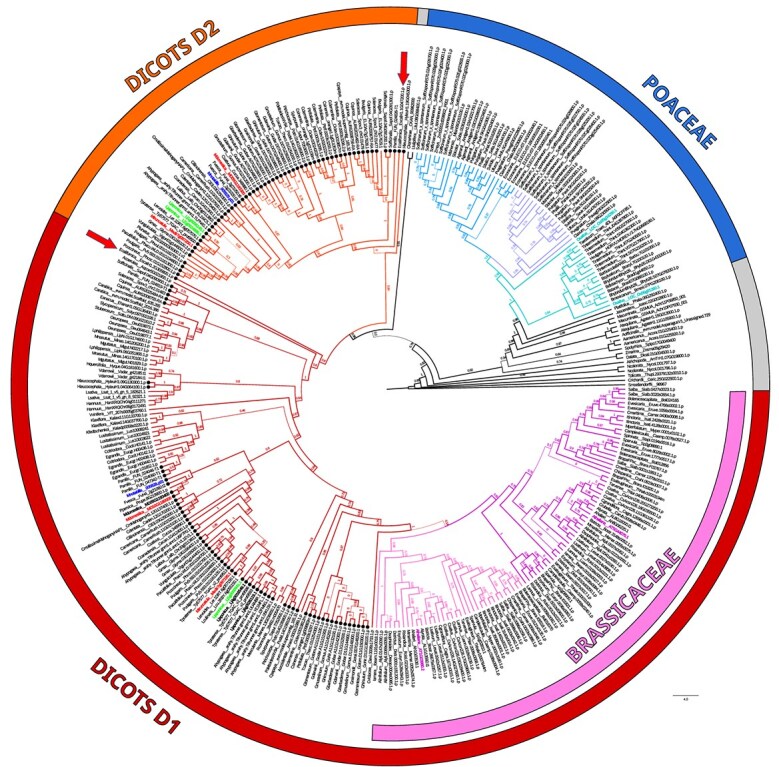
Maximum likelihood cladogram of PCS proteins from fully-sequenced tracheophyte genomes in Phytozome 13. The labels of the cladogram are composed of the abbreviated name of the species and the corresponding protein accession. The circular cladogram is rooted at *Selaginella moellendorffii* Hieron. The external arcs correspond to the three major duplications identified: D (dicotyledons; divided in the two sub-branches D1 and D2); B (Brassicaceae); P (Poaceae). The thickness of the branches is proportional to the SH-like branch support values reported at the base of each clade. The protein labels in bold red correspond to the four PCS characterized in this work. The protein labels in other bold colors correspond to selected PCS proteins characterized in previous works (*Arabidopsis* AtPCS1 and AtPCS2; rice OsPCS1 and OsPCS2; *L. japonicus* LjPCS1, LjPCS2 and LjPCS3; *M. notabilis* MnPCS1 and MnPCS2). The protein labels in bold black correspond to PCS proteins from the same species of PCS characterized in previous works, but still not functionally characterized. The circles at the end of each leaf of the cladogram indicate proteins present in both D1 and D2 branches of the D duplication. Arrows indicate the two PCS from *E. californica*, which belong to the earliest branching order (Ranunculales) of the D duplication.

We performed an extensive search for PCS homologs in the fully sequenced genomes of tracheophytes present in Phytozome 13 using AtPCS1 as a query for BlastP searches. Variable numbers of putatively functional *PCS* genes were identified in 136 fully sequenced genomes of tracheophytes, three of which from Polypodiopsida, Lycopodiopsida, and Pinopsida were used as outgroups with respect to angiosperms (Magnoliopsida and Liliopsida). *Selaginella moellendorffii* Hieron. and the two other outgroup species from Polypodiopsida and Pinopsida (*Ceratopteris richardii* Brongn. and *Thuja plicata* Donn ex D.Don, respectively), had each a single *PCS* gene per genome. In angiosperms, the average number of gene copies per genome was 2.5, but in several genomes *PCS* gene fragments and probable pseudogenes lacking one or more of the amino acids of the catalytic triad were found. The maximum likelihood tree of the resulting dataset is relatively well resolved and overall consistent with the known taxonomy of the species. Three major PCS duplications are evident. One (from now on called the P duplication) is within the family Poaceae, with a relatively complex structure indicating that it could be the result of a first duplication encompassing all Poaceae, followed by the duplication already reported for the tribe Panicoideae [23]. The two rice PCS paralogs are basal with respect to the Panicoideae-specific duplication and the rest of Poaceae, suggesting that they could be the only remnant of the first duplication (Fig. 1).

The second duplication, called D, encompasses many dicotyledon families. It has two branches of different sizes, the largest one indicated as D1, while the other one as D2 (Fig. 1). The presence of one *Escholzia californica* Cham. (Papaveraceae) PCS paralog at the base of both the D1 and D2 branches suggests that the D duplication happened early in the radiation of angiosperms, during or before the evolution of Ranunculales, close to the radiation of the core eudicots (See Fig. 1). By mapping on the accepted phylogeny established in the frame of APG4 [26] the orders with species belonging to the D duplication, it appears evident that one or the other of the paralogs have been lost in many early branching orders of dicots especially in the D2 branch (Fig. S1), suggesting that possible fitness trade-offs or genome evolutionary forces makes the duplication of *PCS* genes a relatively labile trait in angiosperms. The orders involved in the D duplication are all apart from Ranunculales belonging to the Pentapetalae clade, including several orders from rosids and asterids. Worth of note, within the D1 clade a nested Brassicaceae-specific duplication (BR) is evident, which encompasses the majority of the 30 species of Brassicaceae included in the analysis.

### Isolation of D1- and D2-clade paralogs of *PCS* genes from *M. domestica* and *M. truncatula*

To test whether functional differences exist between PCS from the D1 and D2 clades and in case if they are clade-specific, initially we attempted to isolate and express in *Escherichia coli* the *PCS* paralogs of both clades from four species: *M. domestica*, *M. truncatula*, *Carica papaya* L., and *Cucumuis sativus* L. Unfortunately, despite systematic attempts in different *E. coli* strains and induction conditions, only the D1 PCS from *C. papaya* (evm.model.supercontig_165.31) and the D2 protein from *C. sativus* (Cucsa.348980) could be obtained as recombinant proteins (data not shown). Only in the case of *M. domestica* and *M. truncatula* were we able to successfully express one paralog per clade, i.e. both PCS proteins present in the *M. truncatula* genome (Medtr7g097190 and Medtr7g097200, corresponding to *MtPCS1* and *MtPCS2*, respectively; Fig. 1) and two out of three *PCS* paralogs from *M. domestica* (MD10G1180000, MD05G1193300, referred to as *MdPCS1* and *MdPCS2*, respectively). A third *PCS* copy from *M. domestica*, MD05G1193400, which we did not manage to amplify, is 94.3% identical at the amino acid level with MD10G1180000 (D1 clade), suggesting that MD10G1180000 can be considered representative of the D1 clade in the lack of MD05G1193400. We, thus, decided to focus exclusively on the characterization of these four *PCS* paralogs from *M. domestica* and *M. truncatula*. In the case of *M. domestica*, two different transcripts were obtained from *MdPCS2* (MD05G1193300), designated as *MdPCS2* (longer version) and *MdPCS2s* (shorter one). The MdPCS2 protein has 33 additional amino acids at the N-Terminus of the protein that are not present in MdPCS2s (as well as in the majority of the other PCS copies from other species). The rest of the sequences of the two isoforms were perfectly identical (Fig. S2), confirming that *MdPCS2s* and *MdPCS2* are the result of differential transcript splicing from the same locus. All five transcripts were used for further analyses.

### PCS1 and PCS2 recombinant enzymes are activated by different heavy metals in *M. domestica* and *M. truncatula*

**Figure 2:**
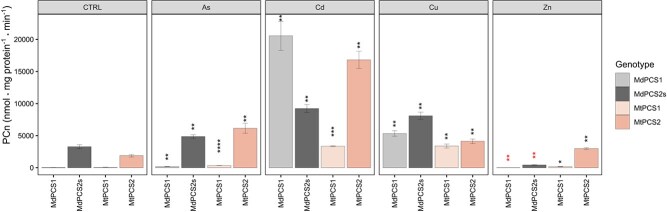
*In vitro* activity of PCn from both the D1 and D2 clades from *M. domestica* and *M. truncatula* in the presence of different metal(loid)s. CTRL corresponds to the basal activity of each of the proteins in the absence of metal(loid)s, while the other panels show the activity of the same proteins challenged with the elements indicated on the top of each panel (As, Cd, Cu, and Zn, respectively). Asterisks indicate significative differences based on Student *t*-test compared to the corresponding protein activity in the CTRL panel, coded according to the following conventions: ^*^  *P* ≤ 0.05; ^**^  *P* ≤ 0.01; ^***^  *P* ≤ 0.001; ^****^  *P* ≤ 0.0001. Asterisks in black indicate values higher than those in control conditions, while red asterisks indicate values lower than those in control conditions.

To evaluate how the different copies of PCSs would respond *in vitro* to different heavy metals, all different copies of PCS identified above from both species were expressed in *E. coli* tagged with 6xHis N-terminal fusions to obtain recombinant proteins to be assayed *in vitro*. Soluble fractions of recombinant proteins were detected and purified for MdPCS1 and MdPCS2s from different *E. coli* strains but failed for MdPCS2, although different *E. coli* strains with combinations of several induction conditions per strain were applied. Consistently with the results for the different copies of MdPCS, the soluble fraction of MtPCS1 and MtPCS2 could be purified by expressing the respective CDS in different *E. coli* strains (see Materials and Methods, Fig. S3). Therefore, PCn production was evaluated for each of the four recombinant proteins obtained (MdPCS1, MdPCS2s, MtPCS1, and MtPCS2) either in control conditions (CTRL) or after exposure to different heavy metals (As^3+^, Cd^2+^, Cu^2+^, and Zn^2+^; Fig. 2). This analysis indicated that in most cases PCn production catalyzed by these proteins was significantly increased to different degrees compared with the corresponding production in control conditions. In all cases the concentrations of metal(loid)s used promoted PCn production, except for a significant repression by the addition of the Zn^2+^ concentration used in this study for both copies of MdPCS.

Taken together, these results imply that both copies of PCS in both species are modulated by all the four metal(loid)s tested. In addition, it was clearly shown that cadmium is the strongest activator for both copies of PCS in both *M. domestica* and *M. truncatula* (Fig. 2). The second strongest activator of both copies of PCS is copper, while activation by arsenic is much stronger for copy 2 than copy 1 of both *M. domestica* and *M. truncatula* (Fig. 2). Zinc among all metal(loid)s tested has a rather weak activation capacity only for MtPCS1 and MtPCS2 proteins.

### D2-clade PCSs complement more effectively the *cad1–3* mutation than their D1 counterparts in both *M. domestica* and *M. truncatula*

**Figure 3:**
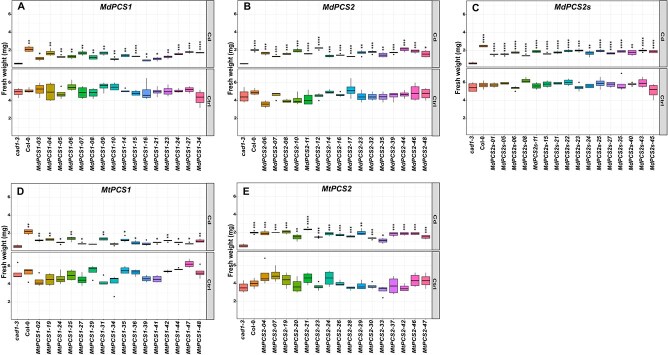
Box plots showing the complementation of the *cad1–3* mutant with different *PCS* genes from *M. domestica* (A–C) and *M. truncatula* (D–E). Each column represents a different genotype either treated (Cd) or not treated (Ctrl) with cadmium. *cad1–3* is the negative control, i.e. the *pcs1* mutant background being complemented. Col-0 is the positive control (*PCS1* background). All the other columns are independent single-copy complementation lines obtained with transgenic *MdPCS1* (A), *MdPCS2* (B), *MdPCS2s* (C), *MtPCS1* (D), and *MtPCS2* (E). Asterisks indicate significative differences based on Student *t*-test compared to the *cad1–3* mutant, coded according to the following conventions: ^*^  *P* ≤ 0.05; ** *P* ≤ 0.01; *** *P* ≤ 0.001; ^****^  *P* ≤ 0.0001.

To elucidate the differential heavy metal responses of different copies of PCSs from both species *in vivo*, three different copies of *PCS* genes from *M. domestica* and two from *M. truncatula* driven by the *AtPCS1* promoter were used to transform the *AtPCS1* knockout mutant *cad1–3* plants. First, 16 independent single-copy transgenic lines per construct were selected based on Kanamycin resistance and used to evaluate the transcription of each *PCS* transgene by semiquantitative reverse transcription polymerase chain reaction (rt-PCR). This analysis showed that each *PCS* gene was expressed in all transgenic complementation lines, although the expression level varied (Fig. S4). Therefore, these transgenic lines were exposed to 8 μM Cd^2+^, the strongest activator based on the enzymatic assays of different copies of PCS *in vitro*. Under control conditions, all tested complementation lines of *PCS* genes from *M. domestica* had similar biomass, but both *PCS* copies partially complemented Cd sensitivity in the *cad1–3* mutant background (Fig. 3).

This analysis further indicated that both *M. domestica* versions of *PCS2* (*MdPCS2* and *MdPCS2s*) derived from differential splicing were not only functional but also shared an analogous function *in vivo* being both more active than *MdPCS1*, as indicated by the higher fresh weight of both the *MdPCS2* complementation lines (*t*-test, *P* = 8.6 × 10^−3^ and *P* = 3.7 × 10^−5^, respectively; Fig. S5). Similarly, all examined complementation lines of both copies of *PCS* genes from *M. truncatula* also shared similar fresh weight under non-treated conditions. However, the *MtPCS1* transgenic lines could complement Cd sensitivity in the *cad1–3* mutant less effectively than *MtPCS2* and a few *MtPCS1* lines did not complement the *cad1–3* mutation (Fig. 3). In fact, the average fresh weight per *MtPCS1* line was significantly lower than that of *MtPCS2* (*t*-test, *P* = 2.2 × 10^−8^; Fig. S5).

**Figure 4:**
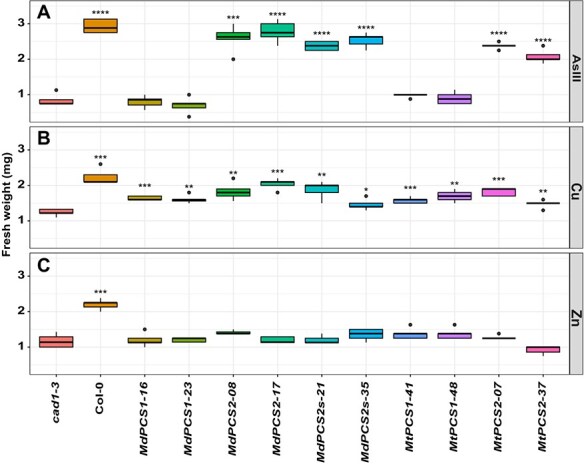
Box plots showing the complementation of the *cad1–3* mutant with selected transgenic lines harboring different *PCS* genes from *M. domestica* and *M. truncatula* treated with different metal(loid)s (A: AsIII; B: Cu; C: Zn). Each column represents the fresh weight of a different genotype treated with the metal(loid) indicated to the right. *cad1–3* is the negative control, i.e. the *pcs1* mutant background being complemented. Col-0 is the positive control (*PCS1* background). All the other columns are independent single-copy complementation lines obtained with transgenic *MdPCS1*, *MdPCS2*, *MdPCS2s*, *MtPCS1*, and *MtPCS2* selected based on the results shown in Fig. 3. Asterisks indicate significative differences based on Student *t*-test compared to the *cad1–3* mutant, coded according to the following conventions: ^*^  *P* ≤ 0.05; ^**^  *P* ≤ 0.01; ^***^  *P* ≤ 0.001; ^****^  *P* ≤ 0.0001.

Based on the Cd responses of these transgenic complementation lines, two representative lines for each PCS transformant were used further to test the effect of exposure to different metal(loid)s (1.2 μM As^3+^, 6 μM Cu^2+^, and 80 μM Zn^2+^). Only *PCS2* from both species turned out to be able to complement As sensitivity in *cad1–3* mutant to levels comparable to Col-0 WT plants, while both *PCS* copies partially complemented Cu sensitivity in the *cad1–3* mutant background and none of the *PCS* copies rescued Zn sensitivity in *cad1–3* mutant complementation tests (Fig. 4).

### D1- and D2-clade PCS differentially regulate PCn and GSH pools in roots and shoots in response to different metal(loid)s

**Figure 5:**
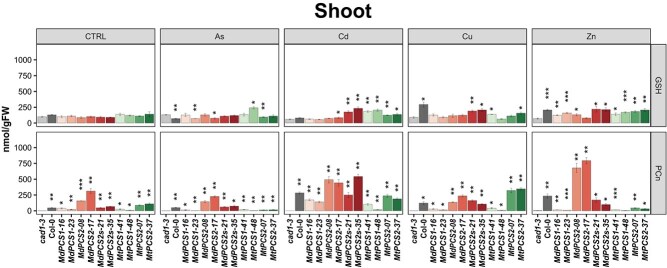
Bar plots showing the content of GSH (upper series of panels) and PCn (lower series of panels) of shoots of the *cad1–3* mutant complemented with selected transgenic lines harboring different *PCS* genes from *M. domestica* and *M. truncatula* treated with different metal(loid)s. Each column represents a different genotype treated with the metal(loid) indicated to the top of each panel (AsIII, Cd, Cu, or Zn). CTRL indicates no application of metal(loid)s. *cad1–3* is the negative control, i.e. the *pcs1* mutant background being complemented. Col-0 is the positive control (WT *PCS1* background). All the other columns are independent single-copy complementation lines obtained with transgenic *MdPCS1*, *MdPCS2*, *MdPCS2s*, *MtPCS1*, and *MtPCS2* selected based on the results shown in Fig. 3. Asterisks indicate significative differences based on Student *t*-test compared to the *cad1–3* mutant, coded according to the following conventions: ^*^  *P* ≤ 0.05; ^**^  *P* ≤ 0.01; ^***^  *P* ≤ 0.001; ^****^  *P* ≤ 0.0001.

**Figure 6:**
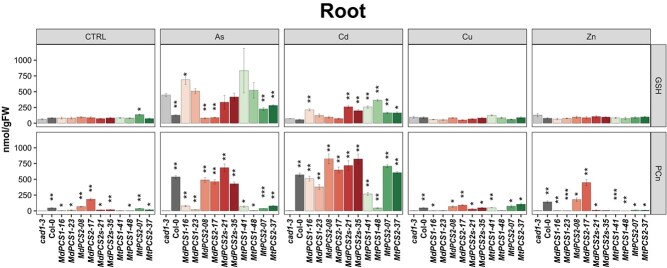
Bar plots showing the content of GSH (upper series of panels) and PCn (lower series of panels) of roots of the *cad1–3* mutant complemented with selected transgenic lines harboring different *PCS* genes from *M. domestica* and *M. truncatula* treated with different metal(loid)s. Each column represents a different genotype treated with the metal(loid) indicated to the top of each panel (AsIII, Cd, Cu, or Zn). CTRL indicates no application of metal(loid)s. *cad1–3* is the negative control, i.e. the *pcs1* mutant background being complemented. Col-0 is the positive control (WT *PCS1* background). All the other columns are independent single-copy complementation lines obtained with transgenic *MdPCS1*, *MdPCS2*, *MdPCS2s*, *MtPCS1*, and *MtPCS2* selected based on the results shown in Fig. 3. Asterisks indicate significative differences based on Student *t*-test compared to the *cad1–3* mutant, coded according to the following conventions: ^*^  *P* ≤ 0.05; ^**^  *P* ≤ 0.01; ^***^  *P* ≤ 0.001; ^****^  *P* ≤ 0.0001.

To investigate the effects of heavy metals on the GSH pool size and production of PCn, total GSH and PCn in shoots (Fig. 5) and roots (Fig. 6) were independently analyzed from different copies of *PCS* complementation lines under different heavy metal treatments. Zn and to lesser extent Cd were the heavy metals causing the largest variation of the GSH pool in the shoots, while the GSH pool had the smallest change in the presence of As and no significant differences were detected under the control condition for GSH in shoots of different copies of *PCS* complementation lines (Fig. 5, upper row of panels).

By contrast, the PCn production in shoots of different copies of *PCS* complementation lines were significantly higher compared with *cad1–3* mutants not only in the control conditions but also in the presence of As, Cu, and Zn, but Cd had overall the largest effect on the induction of PCn production in the shoots. *PCS2* from both species tended to have higher activity than *PCS1* either constitutively or in the presence of Cd. *MdPCS2* had the highest responsiveness to Zn, while *MtPCS2* was the most responsive toward Cu among the PCS genes studied (Fig. 5, lower row of panels).

In parallel, root GSH and PCn levels in response to different metal(loid) treatments of *Arabidopsis* transgenic plants complemented with *PCS* genes from *M. domestica* or *M. truncatula* were also evaluated. Cd tended to increase and As to decrease the GSH pool in the roots compared to the *cad1–3* mutant background in the same conditions (Fig. 6, upper row of panels). Cd and to a lesser extent As had the largest effect on the induction of PCn production in the roots. *PCS2* genes from both species tended to have higher activity than *PCS1* genes in the presence of Cd. Analogously to what was observed in the shoot, *MdPCS2* was also the most responsive gene to Zn and had a higher constitutive activity compared to all other genes (Fig. 6, upper row of panels).

### D1- and especially D2-clade PCS effectively prevent As but not Cd accumulation in shoots

**Figure 7:**
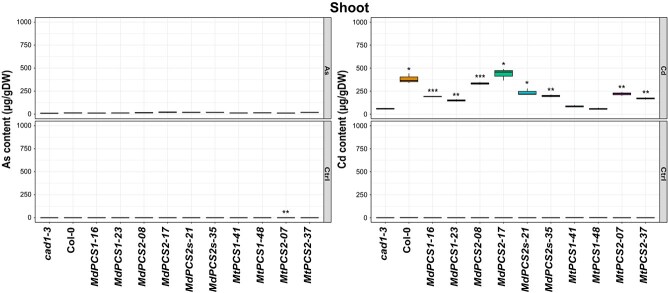
Box plots representing the content of As (left panel) and Cd (right panel) of shoots of the *cad1–3* mutant complemented with selected transgenic lines harboring different *PCS* genes from *M. domestica* and *M. truncatula* treated with different metal(loid)s. Each column indicates a different genotype treated with the metal(loid) shown to the right side of each panel (AsIII or Cd). CTRL indicates no application of metal(loid)s. *cad1–3* is the negative control, i.e. the *pcs1* mutant background being complemented. Col-0 is the positive control (WT *PCS1* background). All the other columns are independent single-copy complementation lines obtained with transgenic *MdPCS1*, *MdPCS2*, *MdPCS2s*, *MtPCS1*, and *MtPCS2* selected based on the results shown in Fig. 3. Asterisks indicate significative differences based on Student *t*-test compared to the *cad1–3* mutant, coded according to the following conventions: ^*^  *P* ≤ 0.05; ^**^  *P* ≤ 0.01; ^***^  *P* ≤ 0.001.

**Figure 8:**
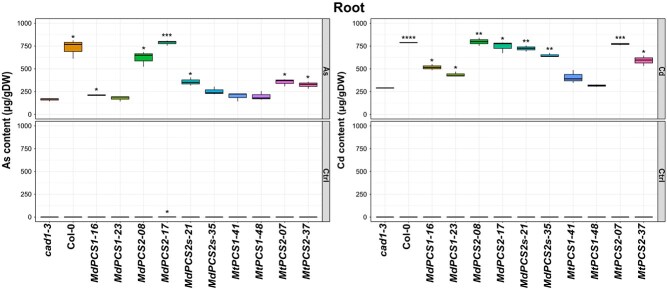
Box plots representing the content of As (left panel) and Cd (right panel) of roots of the *cad1–3* mutant complemented with selected transgenic lines harboring different *PCS* genes from *M. domestica* and *M. truncatula* treated with different metal(loid)s. Each column indicates a different genotype treated with the metal(loid) shown to the right side of each panel (AsIII or Cd). CTRL indicates no application of metal(loid)s. *cad1–3* is the negative control, i.e. the *pcs1* mutant background being complemented. Col-0 is the positive control (WT *PCS1* background). All the other columns are independent single-copy complementation lines obtained with transgenic *MdPCS1*, *MdPCS2*, *MdPCS2s*, *MtPCS1*, and *MtPCS2* selected based on the results shown in Fig. 3. Asterisks indicate significative differences based on Student *t*-test compared to the *cad1–3* mutant, coded according to the following conventions: ^*^  *P* ≤ 0.05; ^**^  *P* ≤ 0.01; ^***^  *P* ≤ 0.001.

To explore whether heavy metal exposure of different copies of *PCS* complementation lines could affect differentially metal(loid) accumulation in shoots and roots, 10-day-old *PCS* complementation lines were subjected to 3-day treatment with 5 μM As^3+^ or 16 μM Cd^2+^, respectively. In the presence of As^3+^ all tested complementation lines of *PCS* genes from both species had similar content of As in shoots, and no significant differences were observed compared with that of *cad1–3*. But in the case of Cd^2+^ exposure, Cd was highly accumulated on both copies of *MdPCS* with a tendency of higher accumulation in *MdPCS2* and *MdPCS2s* compared with *MdPCS1*, and only significantly increased in *MtPCS2* shoots instead of *MtPCS1* shoots (Fig. 7). Similarly, *PCS2* complementation lines in both species accumulated significantly more As in roots, and Cd was also significantly stored in both copies of *MdPCS* roots and in *MtPCS2* roots relative to that of *cad1–3* (Fig. 8).

Two putative D1–D2 specificity-determining positions (SDPs) possibly explaining the observed differences in activity were identified with the help of the S3Det method as implemented in the JDet software [27, 28]. The first position, corresponding to amino acid #47 of *A. thaliana* PCS1, is in the N-terminal domain of PCS. In D1-clade proteins it is highly enriched in phenylalanine, while in D2 proteins the dominant amino acid is tyrosine. The second position identified as a putative SDP is at the beginning of the C-terminal domain of the protein and corresponds to amino acid #270 of AtPCS1. This putative SDP is constituted nearly exclusively by leucine in D2 proteins, while in D1 proteins it mainly consists of more polar amino acids like proline and alanine. Another region resulting from visual inspection of the multiple sequence alignment and specifically found in D1 clade PCS paralogs is a stretch of amino acids present between positions 471 and 478 of AtPCS1, which is absent from D2 clade proteins.

## Discussion

Despite the undisputed relevance of PCS for metal detoxification and homeostasis in both vascular and non-vascular plants [3, 29–33] and the considerable number of fully sequenced, high-quality crop plant genomes available [25], until now no overview of the patterns of *PCS* gene evolution has been reported for land plants. The large-scale genome analysis we conducted in this study, thus, uncovered for the first time different peculiar features of *PCS* gene evolution in dicotyledons. First, it confirmed that the number of *PCS* genes seems to be constrained [10], as, on average, less than three copies of *PCS* are present per genome in the 143 species analyzed in this study, many of which are among the most important crops worldwide. This is in agreement with the observation that gain-of-function of *PCS* genes in heterologous systems may sometimes result in decreased, rather than increased, tolerance to metal(loid)s [34, 35], suggesting that excessive PCS activity can be counterproductive for plant fitness and yield. The second peculiar feature we uncovered is the tendency of *PCS* genes to undergo duplication, either at the family (i.e. Poaceae and Brassicaceae P and B duplications, respectively) or higher taxonomic distances (i.e. the dicotyledon-specific, ancient D duplication; Fig. 1). Surprisingly, while the most recent, family-specific duplications have been already identified [10, 23], the most ancient D duplication has not been detected as such, with only three previous reports dealing in isolation with the characterization of members of both clades of the D duplication (three genes in *L. japonicus* and two in *M. notabilis*, respectively [16, 17, 36, 37]. It is known that gene copies originated from whole-genome duplication (WGD) tend to be more stable than those originated from small-scale duplication (SSD; [38]). It could be thus legitimate to assume that the D1/D2 duplication may have originated by the γ WGD, which happened at the base of the eudicotyledons [39]. This, however, does not seem the case for the D duplication, as a relatively high number of the D1/D2 pairs are retained as tandem duplications contiguous or nearly so in the respective genomes (see, e.g. *M. domestica* and *M. truncatula* D1 and D2 copies in Fig. 1), an event too unlikely to have originated by chance rearrangements following a WGD. Thus, it is more likely that the D duplication arose as a tandem duplication, followed in many but not all the genomes by further rearrangements that led to loss of microsyntenic order conservation [40].

By contrast to duplicated genes originated by WGD, SSD gene duplications tend to be under-represented in protein–protein interaction networks. This is indeed the case of PCS, for which to date just two putative interactors derived from two independent high-throughput screenings have been described [41, 42], lending further support to a segmental origin of the D duplication. It is noteworthy that PCS has been reported to be a homodimeric protein [43]. While the higher conservation of the N-terminus, whose first two conserved helices have been implicated in the dimerization of NsPCS from *Nostoc* sp. [44], may in principle also support heterodimerization, as of today no evidence in support of such a hypothesis exists in literature. On the contrary, no evidence of cooperativity among homodimeric PCS subunits have been identified [44], indicating that dimerization is likely not the driving force behind long-term PCS paralog retention. Thus, dosage balance intended in the most classical way, i.e. that entailing participation to multiprotein complexes [45], seems not to be the case. However, extensive literature reported a clear dosage effect associated to PCS, which, when overexpressed, often phenocopied loss-of-function mutations [46]. This effect has been attributed to a dosage balance sensitivity between the GSH and PCn pools, being GSH both the PCS substrate for PCn production and an important cell redox agent [47, 48]. Another mechanism may also contribute to the long-term retention of the D1/D2 duplication, namely copy functional specialization [40, 45]. Two of the most common types of copy functional specialization are either through gain of function (neofunctionalization) of at least one of the two copies or loss and repartition of function(s) among them (subfunctionalization; [40]). We, thus, checked whether the retention of both D1 and D2 copies for such a long evolutionary time could be associated with copy-specific functional specializations. Unfortunately, the extensive differences in the heterologous system used for *PCS* copies overexpression (*Saccharomyces cerevisiae*), the impossibility to characterize recombinant *L. japonicus* PCS proteins *in vitro*, and in general the different assay conditions used [16, 37] make the comparison of clade-specific PCS features arduous in Fabaceae. The situation in the case of *M. notabilis* is more amenable to comparison, as also in this case an *in planta* (*A. thaliana*, *Nicotiana tabacum*) assessment of the functional properties of *MnPCS1* and *MnPCS2* have been carried out [17]. However, in the latter study, overexpression of the *PCS* genes driven by the strong viral 35S promoter in heterologous WT backgrounds was used [17], while we took a more subtle approach based on complementation in *Arabidopsis* of the *cad1–3* mutant using the endogenous promoter of the *AtPCS1* gene to drive the expression of the *PCS* copies from *M. domestica* and *M. truncatula*. By employing a high number (16 for each of the five constructs) of independent, single-copy transgenic events we could demonstrate that the potential activity of PCS proteins in a simplified *in vitro* assay tends to differ from that of the actual protein *in vivo*, where the fluxes of the different metalloids can influence each other, and compartmentalization plays an important role in metal detoxification [6, 7]. This has been reported also for the overexpression of *L. japonicus LjPCS1* and *LjPCS3* in *S. cerevisiae*, albeit as mentioned earlier, the limited stability of the LjPCS3 (clade D2) protein *in vivo* and the inactivity of the recombinant proteins hinders the interpretation of the relative differences among gene copies [16].

By comparing the average fresh weight of the complementation lines, we could also show that the PCS from the D2 clade in our study are on average significantly more active than those from the PCS1 clade (Fig. 3 and 4). The *in silico* comparison of the proteins from clades D1 and D2 provided two candidate specificity determining positions that may underly such functional differences among paralogs, one in the N-terminal and the other in the C-terminal domain of the enzyme. While the amino acids preferentially found in the putative N-terminal specificity determining position (phenylalanine and tyrosine, respectively for D1 and D2 proteins) are both aromatic and differ only by the presence of a hydroxyl group, we expect a relatively low impact, if any, on the catalytic activity of the enzyme, which resides in this domain of PCS [3]. On the other hand, the more pronounced physico-chemical differences of the amino acids at the putative C-terminal specificity determining position suggest that the differences observed between D1 and D2 paralogs may stem from modulation of the enzyme’s responsiveness to metal(loid)s mediated by the C-terminal domain [13]. As this region was not associated to any specific metal(loid) in previous studies [15, 32], it is possible that it may mediate the enhanced response of D2 clade paralogs to both cadmium and arsenic. Noteworthy, the stretch of about eight amino acids specifically associated with D1 clade proteins was previously proposed to repress the activation by arsenic in AtPCS1, suggesting that they could be responsible for the higher complementation efficiency, PCn production as well as As(III) accumulation in transgenic Arabidopsis transformed with D2 rather than D1 clade *PCS* genes (Fig. 2, 4, 5, 6 and 8). Confirmation of the roles of these putative specificity determining positions, however, awaits experimental validation.

The difference in D1 and D2 protein activities contrasts with *M. notabilis*, where the MnPCS1 copy (from the D1 clade) had been proposed to confer a slightly higher protective role *in vivo* compared to MnPCS2 (from the D2 clade; [17]). We note, however, that both the overexpression and the lower number of potentially multicopy lines used earlier in the screening for activity provide a lower resolution compared to that attained in our study. This could explain the partial discrepancy we obtained from the comparison of D1 and D2 PCS from *M. domestica* and *M. truncatula* with those from *M. notabilis*. Another possibility is that during the Rosales radiation, *M. notabilis* diverged from the other species in terms of PCS functions. Both *M. domestica* and mulberry belong to the Rosales, albeit to distant families, which encompass the longest divergence within the order Rosales [49], providing ample opportunities for functional divergence.

Another difference between the two PCS we characterized and those previously analyzed in *L. japonicus* is that in our study Cd is for both D1 and D2 clades of PCS the strongest activator *in vivo* and *in vitro* of PCn production (Fig. 2, 5 and 6), implying that Cd detoxification was one of the main functions of the PCS common ancestor of both PCS clades. Recent results in the early diverging land plant, the bryophyte *M. polymorpha*, support and extend this view, suggesting that Cd detoxification was most likely one of the ancestral functions of PCS in the common ancestor of all land plants [50]. In *L. japonicus*, by contrast, both the *in vivo* and the *in vitro* assays using overexpression in yeast indicated Cu and Ag as the most potent activators of PCn production [16], but in the lack of overexpression lines for *LjPCS* in Arabidopsis these results must be taken with caution.

We additionally found that As is a stronger activator of PCS from clade D2 than from clade D1, suggesting that a possible subfunctionalization or neofunctionalization of PCS responsiveness toward As could have taken place after the D duplication. Also in this case, the comparison with *M. polymorpha* is informative, as in the bryophyte lineage a functional system based on the ACR3 H^+^/As^3+^ antiporter specifically dedicated to As detoxification which is independent from PCS exists [51, 52]. Therefore, MpPCS has been shown to be fundamentally unresponsive to As [50], lending support to the hypothesis that the capacity to respond to As^3+^ could be a derived trait in PCS genes from clade D2. Taken together, this evidence suggests that in Rosales and Fabales, the PCS of the D1 and D2 clades have different specializations, and that the function of each paralog tend to be conserved across these two orders. Unfortunately, the difficulties in the isolation and expression of PCS from *C. papaya* and *C. sativus* prevented us from reaching a firmer conclusion about the generality of such functional conservation in other crops. Despite this limitation, the Fabales and Rosales are thought to have diverged shortly after the estimated age of the clade encompassing also the other rosid orders at ~100–90 million years ago [53], suggesting that the specialization of PCS belonging to the D1 and D2 clades could have remained relatively stable for a long evolutionary time during dicotyledon evolution and crop domestication.

In conclusion, it is noteworthy that the PCS functionally characterized most in depth until now, *AtPCS1* and *AtPCS2* as well as *OsPCS1* and *OsPCS2*, have been sampled from two family-specific duplications (Fig. 1). Therefore, it is possible that the characterized PCS could represent clade-specific sub- or neofunctionalizations [40], while the broader evolutionary picture on PCS has been until now largely overlooked. As the PCn detoxification pathway is the most important metal(loid) detoxification discovered to date in tracheophytes, the characterization of clade D2 in additional species will provide deeper insights into the interplay of the two PCS copies in metal(loid) detoxification in crops where the D2 copy might play a significant role in metal(loid) detoxification.

## Materials and methods

### Plant materials, growth conditions, and heavy metal treatments

*Malus domestica* (Suckow) Borkh. *‘*Golden delicious*’, M. truncatula* Gaertn. as well as *A. thaliana* (L.) Heynh. ecotype Col-0 WT, *cad1–3* mutant and transgenic plants in either WT or mutant backgrounds were used in this study. All plants were grown under long-day conditions (16 h light/8 h dark) at 22 ± 1°C with light intensity of 100–120 μmol m^−2^ s^−1^ in a growth chamber. For biomass analyses upon heavy metal treatments in *Arabidopsis* Col-0, *cad1–3* mutant and transgenic plants, seeds sterilized as previously described [54] were sown in modified one-tenth Hoagland medium in 100 × 100 × 15-mm square plates as previously indicated [15]. The plates were supplemented without or with 8.0 μM CdSO_4_, 1.2 μM NaAsO_2_, 6.0 μM CuSO_4_, and 80.0 μM ZnSO_4_ and stratified at 4°C for 3 days, germinated, and grown vertically in the growth chamber for an additional 10 days. At least 40 plants for each genotype in each condition were used for biomass analyses.

### Phylogenetic reconstruction

PCS homologs were identified by BlastP using AtPCS1 as a query with an E-value threshold of 1.0^−10^ against a selection of fully sequenced genomes of tracheophytes from Phytozome 13. A total of 331 putative PCS homologs corresponding to the peptide encoded by the primary transcript with a full catalytic triad were downloaded from a total of 136 species (Supplementary Table S1). For each of the PCS homologs we downloaded also the chromosomal coordinates, which were used together with the locus ID for inferring tandem duplications. A duplication was considered a tandem duplication if the paralogs originated by it were next neighbors in the genomes of most species displaying the duplication. Thus, for instance, for the D1–D2 duplication the D1 paralog of *M. truncatula* is gene number 9719 on chromosome 7 (ID: Medtr7g097190.1), while the D2 paralog is gene number 9720 of the same chromosome (ID: Medtr7g097200.1).

The proteins were renamed and aligned with MAFFT Version 7.467 using default parameters. High-quality regions in the alignment were selected with BMGE v1.12_1 [55] using a sliding window of size 3, maximum entropy threshold 0.5, gap rate cut-off 0.5, minimum block size 5, and matrix BLOSUM62, resulting in the retention of 357 aligned positions. The best protein evolution model fitting the alignment was selected with SMS v1.8.1 [56] and was JTT + G + I + F based on the AIC criterion. A maximum likelihood phylogeny was reconstructed using PhyML v3.0 [57, 58]. The selected equilibrium frequencies used with the JTT evolutionary model were empirically determined from the data, using an estimated proportion of invariable sites of 0.025, four substitution rate categories, and an estimated gamma shape parameter of 1.166. The tree topology was optimized using an SPR-based tree topology search and SH-like branch supports were computed. The resulting tree was imported in FigTree v. 1.4.4 (https://github.com/rambaut/figtree/tree/v1.4.4), re-rooted with *Selaginella moellendorffii* Hieron. and annotated.

### Prediction of putative SDPs

To gain deeper insights into the possible determinants of the functional differences among D1 and D2 paralogs, the amino acidic residues differentially conserved within each duplication were investigated with the S3Det method [28] as implemented in the JDet software [27]. The multiple sequence alignment used for phylogenetic reconstruction was filtered using the following parameters: 24% of minimal sequence identity to the alignment master sequence (Cpapaya__evm.model.supercontig_165.32), 90% of minimal amino acidic identity between any pair of proteins, 65% of minimum coverage with respect to the master sequence, and removal of positions with gaps in the master sequence. The S3Det analysis was run using the -v (verbose) option in supervised mode by attributing each paralog to either the D1 or the D2 clades according to the phylogenetic reconstruction.

### Cloning, plasmid constructs, and transformation

First, a 2.0-kb intergenic region upstream of the *AtPCS1* coding sequence was amplified as formerly described [59] with Phusion High-Fidelity DNA Polymerase (Thermo Scientific) using genomic DNA isolated with the CTAB method from Col-0 plants as template and the primers listed in Supplementary Table S1, which contained the BamHI and KpnI sites. The resulting amplicon was cloned into a pENTR/D TOPO vector (Invitrogen) and the resulting plasmid was named pENTR_AtPCS1prom. Second, the pENTR_AtPCS1prom was digested either with KpnI and AscI restriction enzymes or with BamHI and AscI and the insert was independently ligated with a 3xFlag Tag digested with the corresponding enzymes (resulting in plasmids pENTR_AtPCS1prom-BamKpn_3xFlag and pENTR_AtPCS1prom-Bam_3xFlag, respectively). The 3xFlag Tag was generated as formerly indicated [60] by annealing oligos either linked with KpnI or BamHI sites at 5′ primer end and AscI at 3′ primer end and cloned into the pGEM-T vector. Third, the full-length cDNA of *PCS* genes amplified with Phusion High-Fidelity DNA Polymerase (Thermo Scientific) using the primers listed in Supplementary Table S1 were first A-tailed and then cloned into the pGEM-T vector. Each full-length cDNA was cut out of pGEM-T, either with BamHI alone or with BamHI and KpnI, and ligated into plasmid pENTR_AtPCS1prom-Bam_3xFlag or pENTR_AtPCS1prom-BamKpn_3xFlag digested correspondingly, and these constructs were recombined into the destination vector pK7WG2D1 using LR clonase II (Invitrogen). The pK7WG2D1 vector was derived from the pK7WG2 vector [61] by eliminating the 35S promoter with SpeI and SacI restriction digestions and recircularized. All sequences were confirmed by Sanger sequencing in a 96-capillary 3370XL DNA analyzer (Thermo Scientific).

Transformations of the five constructs into the *cad1–3* mutant and transgenic line screening were performed as previously described [23]. T3 homozygous single-copy seeds were used for all downstream analyses.

### Total RNA isolation, cDNA synthesis, and semiquantitative rt-PCR assays

Total RNA extraction and cDNA synthesis were carried out as previously described [62]. Semiquantitative rt-PCR was conducted using *AtEF1α* as a reference gene for *Arabidopsis* transgenic plants. All primer sequences are listed in Supplementary Table S1.

### Construction of recombinant protein for PCSs and PCS activity analyses

The coding sequences of each full-length PCS protein were amplified from corresponding cDNA with Phusion High-Fidelity DNA Polymerase (Thermo Scientific) and the forward and reverse primers, listed in Supplementary Table S1, which added the BamHI and SalI restriction sites to the 5′ and 3′ end of the amplicons, respectively. After A-tailing with Taq DNA polymerase (sigma), the resulting PCR products were cloned into pGEM-T. The plasmid harboring the correct cDNA sequences were digested with BamHI and SalI and the inserts were cloned into the expression vector pET28a digested with the same restriction enzymes to be in-frame with an N-terminal 6xHis-tag. The expression plasmids were transformed into different *E. coli* strains, namely MdPCS1 in BL21(DE3)Star, MdPCS2S in Rosetta™(DE3), MtPCS1 in Codon Plus, and MtPCS2 in Rosetta™(DE3). The recombinant protein purification, quantification, and PCS activity assay were performed as previously described [23] using the following metal concentrations: 100 μM CdSO_4_, 100 μM NaAsO_2_, 100 μM CuSO_4_, and 200 μM ZnSO_4_.

### Thiol-peptides analyses

Ten-day-old plants (*Arabidopsis* Col-0, *cad1–3* mutant, and transgenic plants) grown vertically in one-tenth Hoagland medium in 100 × 100 × 15-mm square plates as mentioned above in the growth chamber under standard long-day condition were transferred to fresh plates supplemented without or with either 5 μM NaAsO_2_, or 16 μM CdSO_4_, or 60 μM CuSO_4_, or 400 uM ZnSO_4_ for 3 days. Aerial parts and roots were collected separately in liquid nitrogen and stored at −80°C till further analyses.

Thiol-peptides analyses and quantification for these samples were conducted as formerly described [63]. At minimum, three biological replicates were used for this analysis.

### Elemental analyses

Ten-day-old plants (*Arabidopsis* Col-0, *cad1–3* mutant and transgenic plants) were grown and treated in the same way as for thiol-peptides analyses, but in this case the harvested shoots and roots were treated with a series of washings as mentioned by Uraguchi et al. [15]. Afterwards, the oven-dried materials were used for elemental analyses as formerly described [50, 52].

### Statistical analyses

Statistical significance among measurements’ means was calculated with Student’s *t*-tests carried out for each measurement versus the respective control (untreated control or WT genotype, depending on the experiment). When more than four tests were carried out for the same family of measurement in one experiment, the false discovery rate (fdr) correction for multiple testing was applied and the corrected *P*-value reported. Unless otherwise specified, the number of stars was proportional to the statistical significance level according to the coding: ^*^P ≤ 0.05; ^**^P ≤ 0.01; ^***^P ≤ 0.001; ^****^P ≤ 0.0001. All experiments were performed with at least *n* = 3 biological replicates.

## Supplementary Material

Web_Material_uhae334

## Data Availability

The data that support the findings of this study are available in the supplementary material of this article.

## References

[1] Clemens S . Evolution and function of phytochelatin synthases. J Plant Physiol. 2006;163:319–32 16384624 10.1016/j.jplph.2005.11.010

[2] Fasani E, Li M, Varotto C. et al. Metal detoxification in land plants: from bryophytes to vascular plants. State of the art and opportunities. Plants. 2022;11:237 35161218 10.3390/plants11030237 PMC8837986

[3] Rea PA . Phytochelatin synthase: of a protease a peptide polymerase made. Physiol Plant. 2012;145:154–64 22224506 10.1111/j.1399-3054.2012.01571.x

[4] Grill E, Winnacker E-L, Zenk MH. Phytochelatins: the principal heavy-metal complexing peptides of higher plants. Science. 1985;230:674–6 17797291 10.1126/science.230.4726.674

[5] Vatamaniuk OK, Mari S, Lu YP. et al. Mechanism of heavy metal ion activation of phytochelatin (PC) synthase. Blocked thiols are sufficient for PC synthase-catalyzed transpeptidation of glutathione and related thiol peptides. J Biol Chem. 2000;275:31451–9 10807919 10.1074/jbc.M002997200

[6] Park J, Song WY, Ko D. et al. The phytochelatin transporters *AtABCC1* and *AtABCC2* mediate tolerance to cadmium and mercury. Plant J. 2012;69:278–88 21919981 10.1111/j.1365-313X.2011.04789.x

[7] Song WY, Park J, Mendoza-Cózatl DG. et al. Arsenic tolerance in *Arabidopsis* is mediated by two ABCC-type phytochelatin transporters. Proc Natl Acad Sci USA. 2010;107:21187–92 21078981 10.1073/pnas.1013964107 PMC3000282

[8] Howden R, Goldsbrough PB, Andersen CR. et al. Cadmium-sensitive, *cad1* mutants of *Arabidopsis thaliana* are phytochelatin deficient. Plant Physiol. 1995;107:1059–66 7770517 10.1104/pp.107.4.1059 PMC157237

[9] Hématy K, Lim M, Cherk C. et al. Moonlighting function of *phytochelatin synthase1* in extracellular defense against fungal pathogens. Plant Physiol. 2020;182:1920–32 31992602 10.1104/pp.19.01393 PMC7140922

[10] Kühnlenz T, Schmidt H, Uraguchi S. et al. *Arabidopsis thaliana* phytochelatin synthase 2 is constitutively active *in vivo* and can rescue the growth defect of the PCS1-deficient *cad1-3* mutant on Cd-contaminated soil. J Exp Bot. 2014;65:4241–53 24821959 10.1093/jxb/eru195 PMC4112630

[11] Lee S, Kang BS. Expression of *Arabidopsis phytochelatin synthase 2* is too low to complement an AtPCS1-defective *Cad1-3* mutant. Mol Cells. 2005;19:81–7 15750344

[12] Li M, Barbaro E, Bellini E. et al. Ancestral function of the phytochelatin synthase C-terminal domain in inhibition of heavy metal-mediated enzyme overactivation. J Exp Bot. 2020a;71:6655–69 32936292 10.1093/jxb/eraa386 PMC7586750

[13] Ruotolo R, Peracchi A, Bolchi A. et al. Domain organization of phytochelatin synthase. Functional properties of truncated enzyme species identified by limited proteolysis. J Biol Chem. 2004;279:14686–93 14729665 10.1074/jbc.M314325200

[14] Vestergaard M, Matsumoto S, Nishikori S. et al. Chelation of cadmium ions by phytochelatin synthase: role of the cystein-rich C-terminal. Anal Sci. 2008;24:277–81 18270423 10.2116/analsci.24.277

[15] Uraguchi S, Sone Y, Ohta Y. et al. Identification of C-terminal regions in *Arabidopsis thaliana* phytochelatin synthase 1 specifically involved in activation by arsenite. Plant Cell Physiol. 2018;59:500–9 29281059 10.1093/pcp/pcx204

[16] Ramos J, Naya L, Gay M. et al. Functional characterization of an unusual phytochelatin synthase, *LjPCS3*, of *Lotus japonicus*. Plant Physiol. 2008;148:536–45 18614711 10.1104/pp.108.121715 PMC2528106

[17] Fan W, Guo Q, Liu CY. et al. Two mulberry *phytochelatin synthase* genes confer zinc/cadmium tolerance and accumulation in transgenic *Arabidopsis* and tobacco. Gene. 2018;645:95–104 29277319 10.1016/j.gene.2017.12.042

[18] Das N, Bhattacharya S, Bhattacharyya S. et al. Identification of alternatively spliced transcripts of rice *phytochelatin synthase 2* gene *OsPCS2* involved in mitigation of cadmium and arsenic stresses. Plant Mol Biol. 2017;94:167–83 28283922 10.1007/s11103-017-0600-1

[19] Hayashi S, Kuramata M, Abe T. et al. Phytochelatin synthase *OsPCS1* plays a crucial role in reducing arsenic levels in rice grains. Plant J. 2017;91:840–8 28621830 10.1111/tpj.13612

[20] Li J-C, Guo J-B, Xu W-Z. et al. RNA interference-mediated silencing of *phytochelatin synthase* gene reduce cadmium accumulation in rice seeds. J Integr Plant Biol. 2007;49:1032–7

[21] Uraguchi S, Tanaka N, Hofmann C. et al. Phytochelatin synthase has contrasting effects on cadmium and arsenic accumulation in rice grains. Plant Cell Physiol. 2017;58:1730–42 29016913 10.1093/pcp/pcx114 PMC5914395

[22] Yamazaki S, Ueda Y, Mukai A. et al. Rice phytochelatin synthases OsPCS1 and OsPCS2 make different contributions to cadmium and arsenic tolerance. Plant Direct. 2018;2:e00034 31245682 10.1002/pld3.34 PMC6508543

[23] Li M, Stragliati L, Bellini E. et al. Evolution and functional differentiation of recently diverged phytochelatin synthase genes from *Arundo donax* L. J Exp Bot. 2019;70:5391–405 31145784 10.1093/jxb/erz266 PMC6793451

[24] Filiz E, Saracoglu IA, Ozyigit II. et al. Comparative analyses of *phytochelatin synthase* (*PCS*) genes in higher plants. Biotechnol Biotechnol Equip. 2019;33:178–94

[25] Goodstein DM, Shu S, Howson R. et al. Phytozome: a comparative platform for green plant genomics. Nucleic Acids Res. 2012;40:D1178–86 22110026 10.1093/nar/gkr944 PMC3245001

[26] The Angiosperm Phylogeny Group, Chase MW, Christenhusz MJM. et al. An update of the angiosperm phylogeny group classification for the orders and families of flowering plants: APG IV. Bot J Linn Soc. 2016;181:1–20

[27] Muth T, García-Martín JA, Rausell A. et al. JDet: interactive calculation and visualization of function-related conservation patterns in multiple sequence alignments and structures. Bioinformatics. 2012;28:584–6 22171333 10.1093/bioinformatics/btr688

[28] Rausell A, Juan D, Pazos F. et al. Protein interactions and ligand binding: from protein subfamilies to functional specificity. Proc Natl Acad Sci. 2010;107:1995–2000 20133844 10.1073/pnas.0908044107 PMC2808218

[29] Bellini E, Bandoni E, Giardini S. et al. Glutathione and phytochelatins jointly allow intracellular and extracellular detoxification of cadmium in the liverwort *Marchantia polymorpha*. Environ Exp Bot. 2023;209:105303

[30] Bellini E, Sorce C, Andreucci A. et al. Intracellular and extracellular thiol-peptides modulate the response of Marchantia polymorphato physiological needs, excess, and starvation of zinc, copper, and iron. Plant Biosyst. 2024;158:754–62

[31] Clemens S, Peršoh D. Multi-tasking phytochelatin synthases. Plant Sci. 2009;177:266–71

[32] Kühnlenz T, Hofmann C, Uraguchi S. et al. Phytochelatin synthesis promotes leaf Zn accumulation of *Arabidopsis thaliana* plants grown in soil with adequate Zn supply and is essential for survival on Zn-contaminated soil. Plant Cell Physiol. 2016;57:2342–52 27694524 10.1093/pcp/pcw148

[33] Petraglia A, De Benedictis M, Degola F. et al. The capability to synthesize phytochelatins and the presence of constitutive and functional phytochelatin synthases are ancestral (plesiomorphic) characters for basal land plants. J Exp Bot. 2014;65:1153–63 24449382 10.1093/jxb/ert472

[34] Lee S, Moon JS, Ko T-S. et al. Overexpression of *Arabidopsis phytochelatin synthase* paradoxically leads to hypersensitivity to cadmium stress. Plant Physiol. 2003a;131:656–63 12586889 10.1104/pp.014118 PMC166841

[35] Lee S, Petros D, Moon JS. et al. Higher levels of ectopic expression of *Arabidopsis phytochelatin synthase* do not lead to increased cadmium tolerance and accumulation. Plant Physiol Biochem. 2003b;41:903–10

[36] Loscos J, Naya L, Ramos J. et al. A reassessment of substrate specificity and activation of phytochelatin synthases from model plants by physiologically relevant metals. Plant Physiol. 2006;140:1213–21 16489135 10.1104/pp.105.073635 PMC1435825

[37] Ramos J, Clemente MR, Naya L. et al. Phytochelatin synthases of the model legume *Lotus japonicus*. A small multigene family with differential response to cadmium and alternatively spliced variants. Plant Physiol. 2007;143:1110–8 17208961 10.1104/pp.106.090894 PMC1820930

[38] Defoort J, Van de Peer Y, Carretero-Paulet L. The evolution of gene duplicates in angiosperms and the impact of protein-protein interactions and the mechanism of duplication. Genome Biol Evol. 2019;11:2292–305 31364708 10.1093/gbe/evz156 PMC6735927

[39] Jiao Y, Wickett NJ, Ayyampalayam S. et al. Ancestral polyploidy in seed plants and angiosperms. Nature. 2011;473:97–100 21478875 10.1038/nature09916

[40] Birchler JA, Yang H. The multiple fates of gene duplications: deletion, hypofunctionalization, subfunctionalization, neofunctionalization, dosage balance constraints, and neutral variation. Plant Cell. 2022;34:2466–74 35253876 10.1093/plcell/koac076 PMC9252495

[41] Arabidopsis Interactome Mapping Consortium . Evidence for network evolution in an *Arabidopsis* interactome map. Science. 2011;333:601–7 21798944 10.1126/science.1203877 PMC3170756

[42] Kim D-Y, Scalf M, Smith LM. et al. Advanced proteomic analyses yield a deep catalog of ubiquitylation targets in *Arabidopsis*. Plant Cell. 2013;25:1523–40 23667124 10.1105/tpc.112.108613 PMC3694690

[43] Rea PA . Phytochelatin synthase. In Rea PA (ed.), Encyclopedia of Life Sciences. Chichester: John Wiley & Sons, Ltd, 2020,1–15

[44] Vivares D, Arnoux P, Pignol D. A papain-like enzyme at work: native and acyl-enzyme intermediate structures in phytochelatin synthesis. Proc Natl Acad Sci USA. 2005;102:18848–53 16339904 10.1073/pnas.0505833102 PMC1310510

[45] Birchler JA, Riddle NC, Auger DL. et al. Dosage balance in gene regulation: biological implications. Trends Genet. 2005;21:219–26 15797617 10.1016/j.tig.2005.02.010

[46] Lee BD, Hwang S. Tobacco *phytochelatin synthase* (*NtPCS1*) plays important roles in cadmium and arsenic tolerance and in early plant development in tobacco. Plant Biotechnol Rep. 2015;9:107–14

[47] Brunetti P, Zanella L, Proia A. et al. Cadmium tolerance and phytochelatin content of *Arabidopsis* seedlings over-expressing the phytochelatin synthase gene *AtPCS1*. J Exp Bot. 2011;62:5509–19 21841172 10.1093/jxb/err228 PMC3223047

[48] Wojas S, Clemens S, Hennig J. et al. Overexpression of *phytochelatin synthase* in tobacco: distinctive effects of *AtPCS1* and *CePCS* genes on plant response to cadmium. J Exp Bot. 2008;59:2205–19 18467325 10.1093/jxb/ern092 PMC2413269

[49] Zhang S, Soltis DE, Yang Y. et al. Multi-gene analysis provides a well-supported phylogeny of Rosales. Mol Phylogenet Evol. 2011;60:21–8 21540119 10.1016/j.ympev.2011.04.008

[50] Li M, Leso M, Buti M. et al. Phytochelatin synthase de-regulation in *Marchantia polymorpha* indicates cadmium detoxification as its primary ancestral function in land plants and provides a novel visual bioindicator for detection of this metal. J Hazard Mater. 2022;440:129844

[51] Dutta P, Prasad P, Indoilya Y. et al. Unveiling the molecular mechanisms of arsenic tolerance and resilience in the primitive bryophyte *Marchantia polymorpha* L. Environ Pollut. 2024;346:123506 38360385 10.1016/j.envpol.2024.123506

[52] Li M, Boisson-Dernier A, Bertoldi D. et al. Elucidation of arsenic detoxification mechanism in *Marchantia polymorpha*: the role of ACR3. J Hazard Mater. 2024;470:134088 38555672 10.1016/j.jhazmat.2024.134088

[53] Wang H, Moore MJ, Soltis PS. et al. Rosid radiation and the rapid rise of angiosperm-dominated forests. Proc Natl Acad Sci. 2009;106:3853–8 19223592 10.1073/pnas.0813376106 PMC2644257

[54] Li M, Yu J, Barbaro E. et al. High-throughput, robust and highly time-flexible method for surface sterilization of *Arabidopsis* seeds. J Vis Exp. 2021;176:e62893 10.3791/62893 34661573

[55] Criscuolo A, Gribaldo S. BMGE (block mapping and gathering with entropy): a new software for selection of phylogenetic informative regions from multiple sequence alignments. BMC Evol Biol. 2010;10:210 20626897 10.1186/1471-2148-10-210 PMC3017758

[56] Lefort V, Longueville JE, Gascuel O. SMS: smart model selection in PhyML. Mol Biol Evol. 2017;34:2422–4 28472384 10.1093/molbev/msx149 PMC5850602

[57] Guindon S, Dufayard JF, Lefort V. et al. New algorithms and methods to estimate maximum-likelihood phylogenies: assessing the performance of PhyML 3.0. Syst Biol. 2010;59:307–21 20525638 10.1093/sysbio/syq010

[58] Guindon S, Gascuel O. A simple, fast, and accurate algorithm to estimate large phylogenies by maximum likelihood. Syst Biol. 2003;52:696–704 14530136 10.1080/10635150390235520

[59] Lee S, Korban SS. Transcriptional regulation of *Arabidopsis thaliana phytochelatin synthase* (*AtPCS1*) by cadmium during early stages of plant development. Planta. 2002;215:689–93 12172853 10.1007/s00425-002-0821-6

[60] Li M, Cappellin L, Xu J. et al. High-throughput screening for in planta characterization of VOC biosynthetic genes by PTR-ToF-MS. J Plant Res. 2020b;133:123–31 31701286 10.1007/s10265-019-01149-z PMC6946754

[61] Karimi M, Inzé D, Depicker A. GATEWAY™ vectors for *Agrobacterium*-mediated plant. Trends Plant Sci. 2002;7:193–5 11992820 10.1016/s1360-1385(02)02251-3

[62] Poli M, Salvi S, Li M. et al. Selection of reference genes suitable for normalization of qPCR data under abiotic stresses in bioenergy crop *Arundo donax* L. Sci Rep. 2017;7:1–11 28878356 10.1038/s41598-017-11019-0 PMC5587670

[63] Bellini E, Borsò M, Betti C. et al. Characterization and quantification of thiol-peptides in *Arabidopsis thaliana* using combined dilution and high sensitivity HPLC-ESI-MS-MS. Phytochemistry. 2019;164:215–22 31177054 10.1016/j.phytochem.2019.05.007

