# Supplementary material for: Ancient duplication and functional differentiation of phytochelatin synthases is conserved in plant genomes
Source: Hortic Res. 2024 Nov 26;12(3):uhae334. doi: 10.1093/hr/uhae334 (PMC11879510; doi:10.1093/hr/uhae334)
Supplement: Web_Material_uhae334 [file web_material_uhae334.zip › Li_et_al_PCS_Evolution_SUPPLEMENTARY_MATERIALS_R01.docx]

**Ancient duplication and functional differentiation of phytochelatin synthases is conserved in plant genomes**

Mingai Li^1,2,*^, Jiamei Yu^1,*^, Silvia Sartore^1^, Erika Bellini^3^, Daniela Bertoldi^5^, Stefania Pilati^1^, Alessandro Saba^6,7^, Roberto Larcher^5^, Luigi Sanità di Toppi^3^, Claudio Varotto^1,2,#^

^1^Biodiversity, Research and Innovation Centre, Fondazione Edmund Mach, via Mach 1, 38098, San Michele all’Adige, Trento, Italy

^2^ NBFC, National Biodiversity Future Center, Palermo 90133, Italy

^3^Dipartimento di Biologia, Università di Pisa, via Luca Ghini 13, 56126, Pisa, Italy

^4^Dipartimento di Biologia e Biotecnologie “Charles Darwin”, Sapienza Università di Roma, Piazzale Aldo Moro 5, 00185, Roma, Italy

^5^ Food Characterization and Processing Department, Technology Transfer Centre, Fondazione Edmund Mach, via Mach 1, 38098 San Michele all’Adige, Trento, Italy

^6^Dipartimento di Patologia Chirurgica, Medica, Molecolare e dell’Area Critica, Università di Pisa, via Roma 67, 56126, Pisa, Italy

^7^Center for Instrument Sharing of the University of Pisa (CISUP), Lungarno Pacinotti, 43/44, 56126, Pisa, Italy

* These authors contributed equally to the work.

**Table S1**. Primers used in this study.

| **Primer name** | **Sequence (5' - 3')** | **Purpose** |
| --- | --- | --- |
| AtPCS1_Prom_For | CACCGAATTTAAGTACATTTCTCATGGGTTGG | Cloning (complementation) |
| AtPCS1_Prom_Rev | GGTACCACCTGGATCCTTTTCACTGCTTGTTTTGGTATCT | Cloning (complementation) |
| Mdom_PCS1_BamF | GGATCCATGGCTATGGCGGGGCTG | Cloning (complementation/recombinant protein) |
| Mdom_PCS1_KpnR | GGTACCAGCGAGAGGGGAGCAAAG | Cloning (complementation) |
| Mdom_PCS1_SalR | GTCGACCTAAGCGAGAGGGGAGCA | Cloning (recombinant protein) |
| Mdom_PCS2_BamF | GGATCCATGGCGGTGGTCGGCCTC | Cloning (complementation/recombinant protein) |
| Mdom_PCS2_KpnR | GGTACCCTTATCAGTCATGAGGAAG | Cloning (complementation) |
| Mdom_PCS2_SalR | GTCGACTCACTTATCAGTCATGAGGAAG | Cloning (recombinant protein) |
| Mtru_PCS1_BamF | GGATCCATGGCGGCGATGGCTGGT | Cloning (complementation/recombinant protein) |
| Mtru_PCS1_KpnR | GGTACCAGAGGAAGGAGCACCGA | Cloning (complementation) |
| Mtru_PCS1_SalR | GTCGACCTAAGAGGAAGGAGCACCGA | Cloning (recombinant protein) |
| Mtru_PCS2_BamF | GGATCCATGGCAATGGCAAGCGCA | Cloning (complementation/recombinant protein) |
| Mtru_PCS2_KpnR | GGTACCGTCAGAACGAGCACCA | Cloning (complementation) |
| Mtru_PCS2_SalR | GTCGACTCAGTCAGAACGAGCACCA | Cloning (recombinant protein) |
| 3xFlag_BamF | GGATCCTCGGATTATAAAGACCATGACG | Cloning |
| Mdom_PCS1_RT_For | GTGGGATGGTGGTAAATGGT | RT-PCR |
| Mdom_PCS1_RT_Rev | GCAAAGTAGGAAGATTTACAGTGG | RT-PCR |
| Mdom_PCS2/2s_RT_For | GTTCCCTCATGTCAAACCGA | RT-PCR |
| Mdom_PCS2/2s_RT_Rev | GTAGCTTCTCTTCTCTAAGACCAG | RT-PCR |
| Mtru_PCS1_RT_For | AAGTGTTTGTTGCCAAGGAG | RT-PCR |
| Mtru_PCS1_RT_Rev | TTGCTCAGTGTTACCATTTACC | RT-PCR |
| Mtru_PCS2_RT_For | GTGGTTGGATTCAGAAAGGTC | RT-PCR |
| Mtru_PCS2_RT_Rev | TTCACTACTACCTCCAGTCGT | RT-PCR |


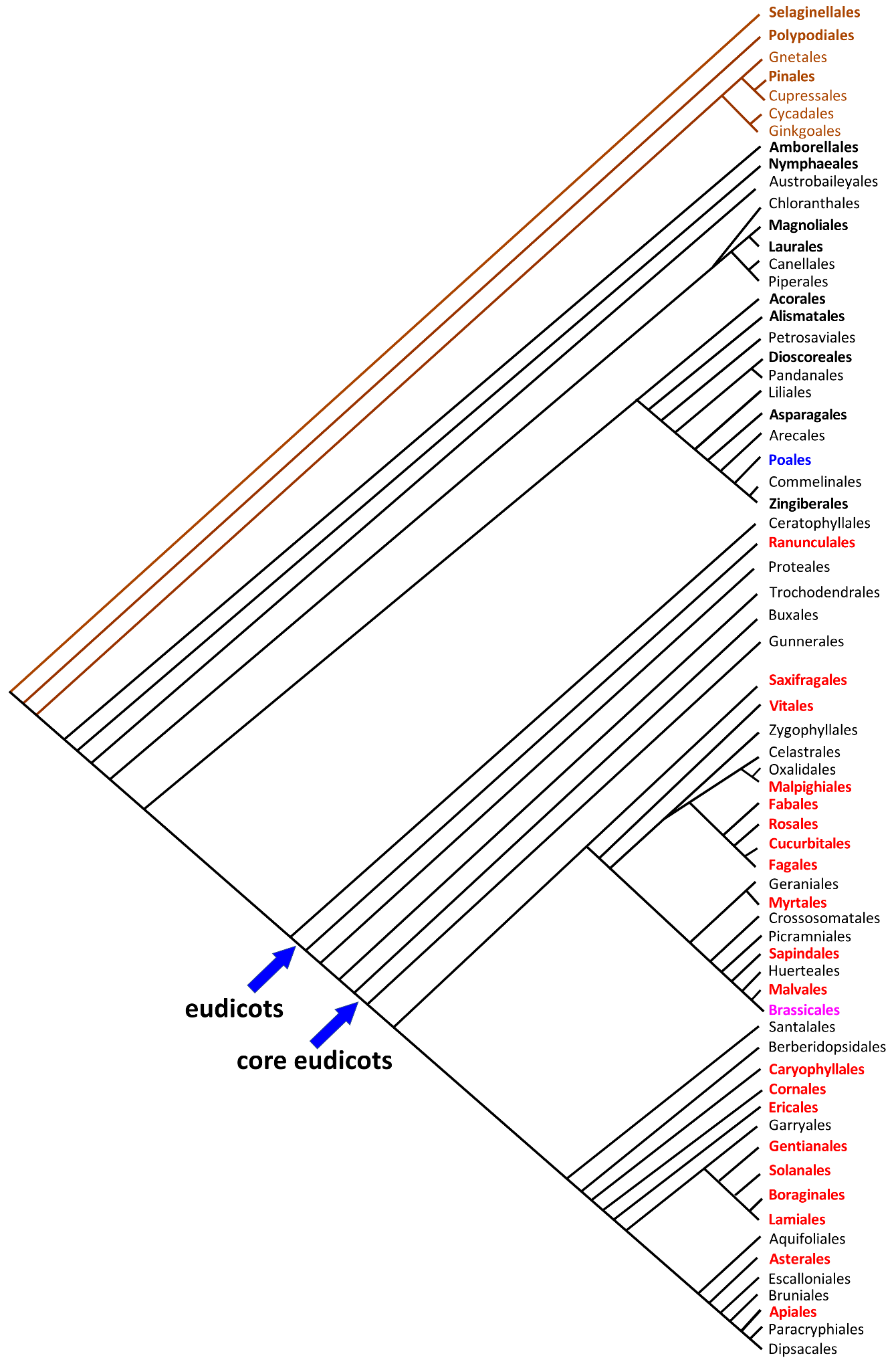


**Figure S1**. Cladogram of the major tracheophyte orders. The names in bold are those of the orders represented by at least one species, while those in normal font are those not represented in Phytozome 13. The names in brown correspond to orders not belonging to angiosperms, and the ones in bold represent the outgroups used for the phylogeny in Fig. 1. The name in bold blue indicates the order of the clade-specific Poaceae duplication (P), while the one in bold pink indicates the order of the clade-specific Brassicaceae duplication (B). The names in bold red indicate the orders with at least one PCS copy in the D clade (D1, D2 or both). The names in bold black are the orders of those genomes where no PCS proteins belonging to the D duplication were found.


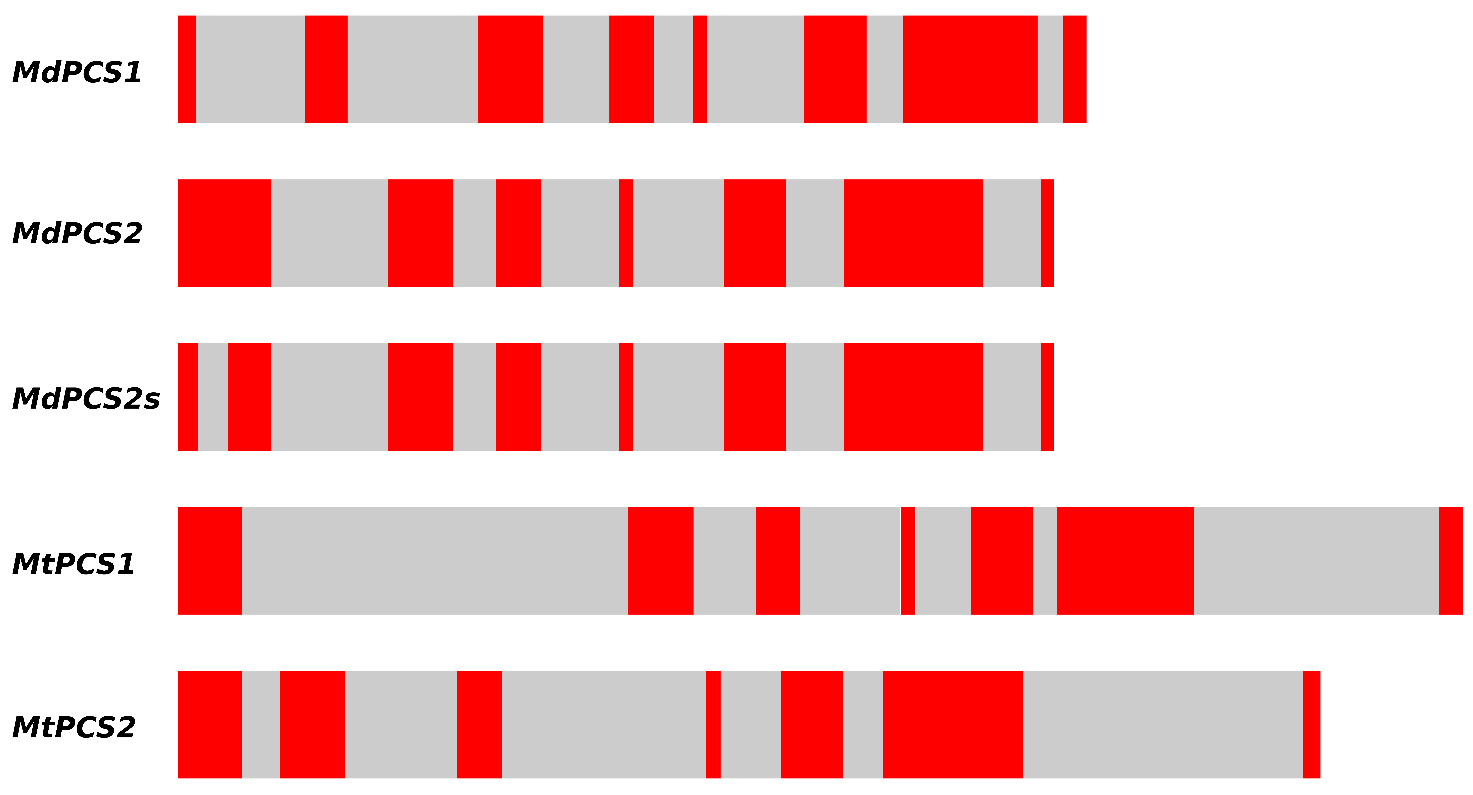


**Figure S2**. Structure of the *PCS* genes from *M. domestica* and *M. truncatula* used for functional analyses. For each gene the exons are shown in red, while introns are shown in gray. The width of exon and introns is drawn to scale to their length in base pairs.

**
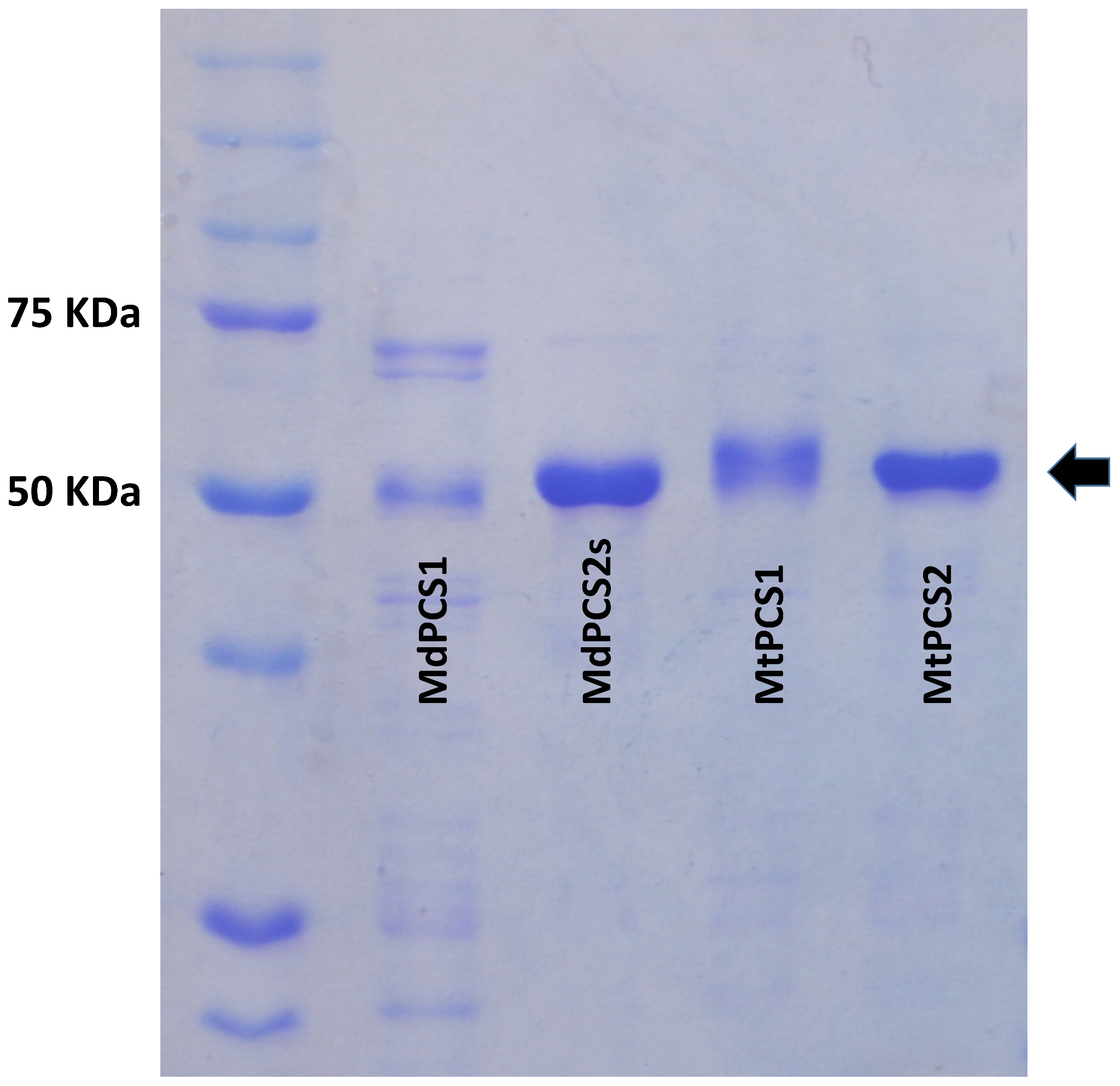
**

**Figure S3**. Recombinant proteins MdPCS1, MdPCS2s, MtPCS1 and MtPCS2 purified from *Escherichia coli* and electrophoresed in 10% SDS-PAGE. The protein size marker is shown in the first lane, two different sizes (50 kDa and 75 kDa) are indicated for the corresponding bands in the marker lane, and the black arrow represents the bands for different PCS proteins.

**
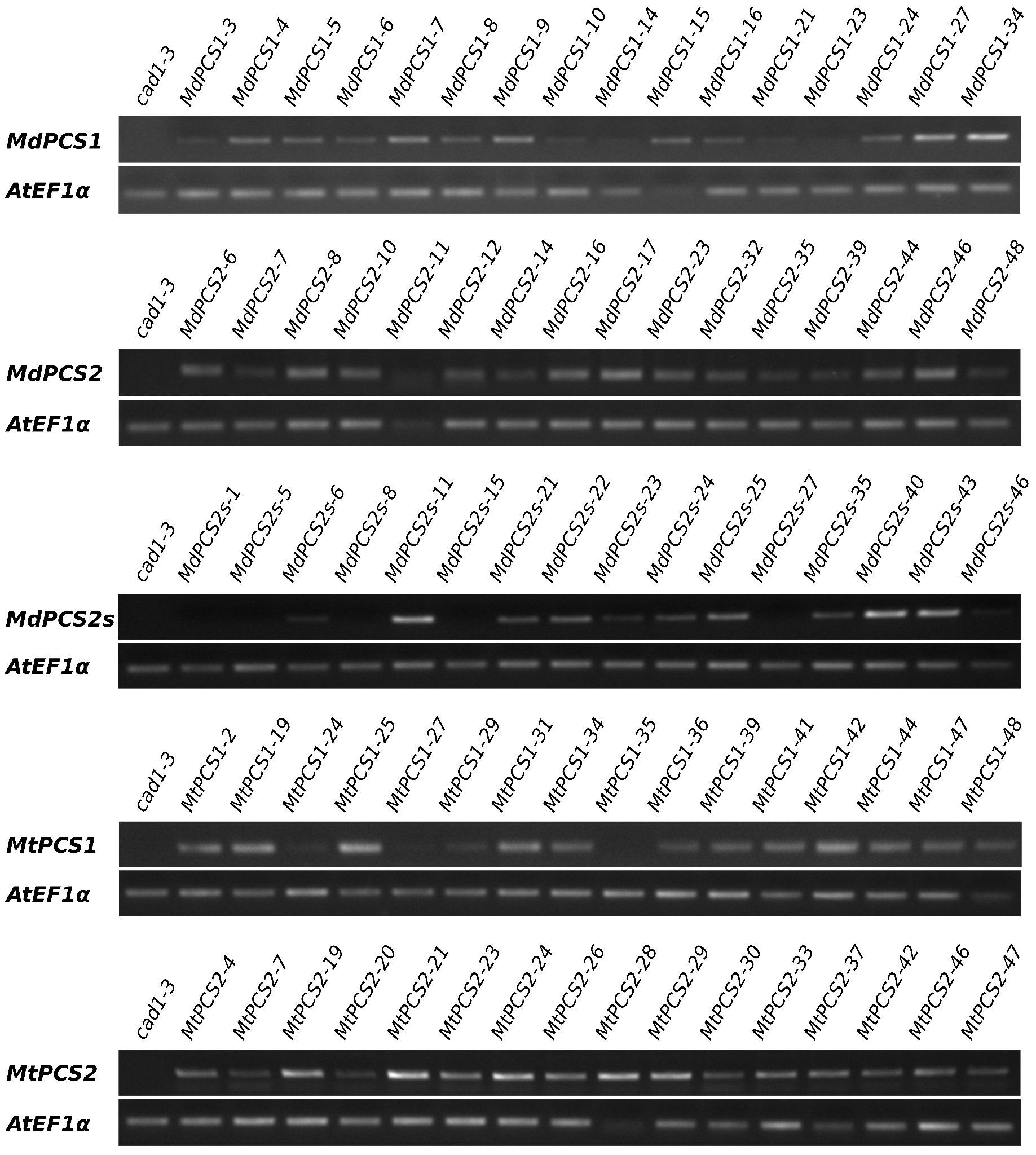
**

**Figure S4.** Semi-quantitative rt-PCR of *MdPCS1*, *MdPCS2*, *MdPCS2s*, *MtPCS1* and *MtPCS2* transcription in 16 independent Arabidopsis transgenic lines. *AtEF1a* was used as an internal reference gene. Thirty-two cycles were used to amplify *MdPCS1, MdPCS2, MtPCS2,* 33 cycles for *MdPCS2s,* 36 cycles for *MtPCS1* and 26 cycles for *AtEF1a*.


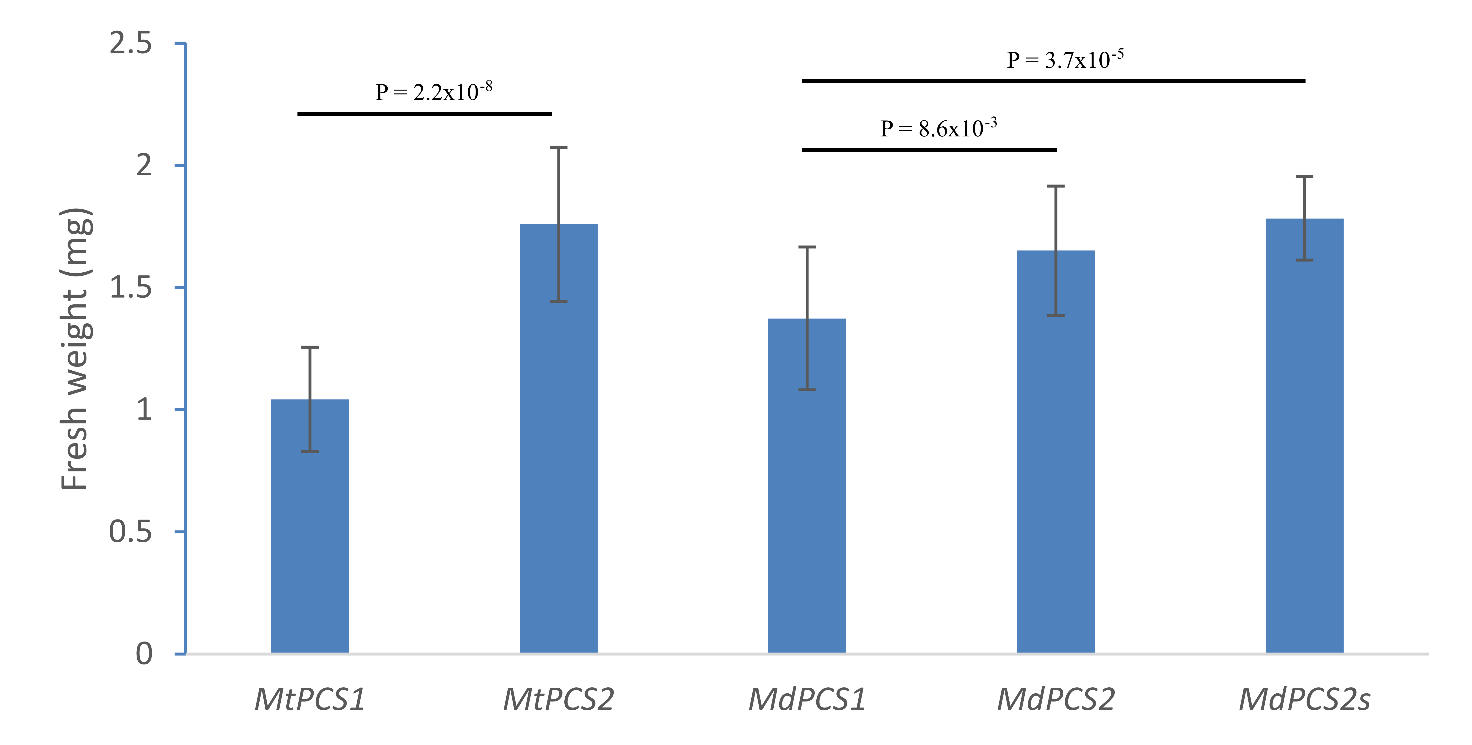


**Figure S5.** Average fresh weight for different *Arabidopsis* *cad1-3* complementation lines treated with cadmium. Each bar represents the average and standard deviation fresh weight for 16 independent single-copy lines transformed with the heterologous *PCS* gene indicated under each bar. The *t*-test probabilities for the comparisons between *PCS* copies 1 and 2 of each species are reported above each pair of columns tested.
